# Medical device management reform, United Republic of Tanzania

**DOI:** 10.2471/BLT.23.290636

**Published:** 2024-07-04

**Authors:** Ally Kebby Abdallah, Suniva Haule, Reinhold Werlein, Valentino Mvanga, Patrick Delcroix, Jasmina Saric, Manfred Stoermer

**Affiliations:** a Health Promotion and System Strengthening Project, Dodoma, United Republic of Tanzania.; b Ministry of Health, Dodoma, United Republic of Tanzania.; c Swiss Tropical and Public Health Institute, Kreuzstrasse 2, 4123 Allschwil, Switzerland.

## Abstract

Health-care technology is central to boosting the productivity and quality of health-care systems. In many sub-Saharan African countries, however, medical device management systems are weak or absent. The aim of this article is to illustrate, using a case study, how policy reforms can help ensure policy on health-care technology is translated into everyday practice and how an integrated systems approach can enhance the operation of medical device management. Between 2011 and 2023, a plan to improve medical device management systems in the United Republic of Tanzania was developed and implemented through Swiss–Tanzanian cooperation within the Health Promotion and System Strengthening Project. The availability of biomedical engineers was increased through new training courses and the creation of permanent positions in government. Moreover, additional district and regional maintenance and repair workshops were built, and a National Centre for Calibration and Training was established to ensure the correct functioning of medical devices. The introduction of an electronic medical device management system provided health facilities and the health ministry with data on the operational status of medical devices and the need for repairs and spare parts. Every level of government was encouraged to allocate more human and financial resources to medical device management. Following this decade-long effort, the percentage of functioning equipment increased substantially, and costs were reduced by repairing rather than replacing equipment. The project also demonstrated the value of an integrated, system-strengthening approach that considered personnel, maintenance and repair facilities, documentation and management, and government policy and budgeting.

## Résumé

Les technologies jouent un rôle crucial dans le renforcement de la productivité et de la qualité des systèmes de santé. Pourtant, dans de nombreux pays d'Afrique subsaharienne, les systèmes de gestion des dispositifs médicaux sont limités, voire inexistants. Cet article a pour but d'illustrer, au moyen d'une étude de cas, comment les réformes peuvent contribuer à faire en sorte que les politiques en matière de technologies sanitaires soient appliquées au quotidien, et comment une approche intégrée peut améliorer la gestion des dispositifs médicaux. Entre 2011 et 2023, un plan visant à développer les systèmes de gestion des dispositifs médicaux en République-Unie de Tanzanie a été défini et mis en œuvre en collaboration avec la Suisse, dans le cadre du Projet de Promotion et de Renforcement du Système de Santé. De nouvelles formations et la création de postes permanents au sein du gouvernement ont permis d'accroître la disponibilité des ingénieurs biomédicaux. En outre, des ateliers supplémentaires d'entretien et de réparation ont été construits dans différentes régions et districts, tandis qu'un Centre National d'Étalonnage et de Formation a ouvert ses portes pour assurer le bon fonctionnement des dispositifs médicaux. L'introduction d'un système électronique de gestion des dispositifs médicaux a fourni aux établissements de soins de santé et au Ministère de la Santé des données concernant le statut opérationnel de ces dispositifs, ainsi que les réparations et pièces détachées requises. Chaque niveau de pouvoir a été encouragé à attribuer davantage de ressources humaines et financières à la gestion des dispositifs médicaux. Au terme de dix ans d'efforts, le pourcentage d'équipements en état de marche a considérablement augmenté et les coûts ont diminué grâce au recours à la réparation plutôt qu'au remplacement. Le projet a également démontré l'importance d'une approche intégrée, qui consiste à renforcer le système en tenant compte du personnel, de l'entretien et des installations de réparation, de la documentation et de la gestion, mais aussi de la politique gouvernementale et du budget.

## Resumen

La tecnología aplicada a la atención sanitaria es fundamental para impulsar la productividad y la calidad de los sistemas sanitarios. Sin embargo, en muchos países del África subsahariana los sistemas de gestión de los productos sanitarios son deficientes o inexistentes. El objetivo de este artículo es ilustrar, mediante un estudio de caso, cómo las reformas políticas pueden ayudar a garantizar que la política sobre tecnología de la atención sanitaria se convierta en una práctica cotidiana y cómo un enfoque de sistemas integrados puede mejorar el funcionamiento de la gestión de los productos sanitarios. Entre 2011 y 2023, se elaboró un plan para mejorar los sistemas de gestión de los productos sanitarios en la República Unida de Tanzania, que se implementó a través de la cooperación suizo-tanzana en el marco del Proyecto de Promoción de la Salud y Fortalecimiento del Sistema. Se aumentó la disponibilidad de ingenieros biomédicos mediante nuevos cursos de formación y la creación de puestos permanentes en el gobierno. Además, se construyeron talleres de mantenimiento y reparación adicionales de distrito y regionales, y se estableció un Centro Nacional de Calibración y Formación para garantizar el correcto funcionamiento de los productos sanitarios. La introducción de un sistema electrónico de gestión de productos sanitarios proporcionó a los centros sanitarios y al Ministerio de Sanidad datos sobre el estado operativo de los productos sanitarios y la necesidad de reparaciones y piezas de repuesto. Se animó a todos los niveles de gobierno a asignar más recursos humanos y financieros a la gestión de los productos sanitarios. Tras este esfuerzo de una década, el porcentaje de equipos en funcionamiento aumentó notablemente y los costes se redujeron al reparar los equipos en lugar de sustituirlos. El proyecto también demostró el valor de un enfoque integrado de refuerzo del sistema que tenía en cuenta el personal, las instalaciones de mantenimiento y reparación, la documentación y la gestión, y la política y los presupuestos gubernamentales.

## ملخص

تعد تكنولوجيا الرعاية الصحية محورًا رئيسيًا للارتقاء بإنتاجية نظم الرعاية الصحية وجودتها. ومع ذلك، فإن نظم إدارة الأجهزة الطبية في العديد من الدول الأفريقية جنوب الصحراء الكبرى، تعد ضعيفة أو منعدمة. الهدف من هذه المقالة هو استخدام دراسة حالة لتوضيح كيف يمكن لإصلاحات السياسة أن تساعد في التحقق من ترجمة سياسة تكنولوجيا الرعاية الصحية إلى ممارسة يومية، وكيف يمكن لأسلوب النظم المتكاملة أن يدعم تشغيل إدارة الأجهزة الطبية. في الفترة بين عامي 2011 و2023، تم وضع خطة لتحسين نظم إدارة الأجهزة الطبية وتنفيذها في جمهورية تنزانيا المتحدة، وذلك من خلال التعاون السويسري التنزاني في إطار مشروع للارتقاء بالصحة ودعم النظام. زاد توافر مهندسي الطب الحيوي من خلال الدورات التدريبية الجديدة، وكذلك استحداث مناصب دائمة في الحكومة. وفضلاً عن ذلك، تم إنشاء ورش صيانة وإصلاح إضافية على مستوى المناطق والأقاليم، وتأسيس مركز وطني للمعايرة والتدريب، وذلك للتحقق من الأداء الصحيح للأجهزة الطبية. إن طرح نظام إلكتروني لإدارة الأجهزة الطبية أدى إلى إمداد المرافق الصحية ووزارة الصحة ببيانات عن حالة تشغيل الأجهزة الطبية، والحاجة إلى الإصلاحات وقطع الغيار. تم تشجيع كل قطاع حكومي على تخصيص المزيد من الموارد البشرية والمالية لإدارة الأجهزة الطبية. وبعد هذا الجهد الذي استمر لمدة عقد من الزمان، زادت النسبة المئوية للمعدات قيد التشغيل بشكل كبير، وانخفضت التكاليف عن طريق إصلاح المعدات بدلاً من استبدالها. وأظهر المشروع أيضًا قيمة الأسلوب المتكامل لدعم النظام، والذي وضع في اعتباره الموظفين، ومرافق الصيانة والإصلاح، والتوثيق والإدارة، والسياسة الحكومية والميزانية.

## 摘要

卫生保健技术对于提高卫生保健系统的生产率和质量至关重要。然而，在撒哈拉沙漠以南的许多非洲国家，医疗器械管理系统仍比较薄弱或根本不存在。本文旨在通过案例研究说明政策改革可如何帮助确保将医疗保健技术相关政策落实到日常实践中，以及综合系统方法可如何改进医疗器械管理系统的运行。2011 年至 2023 年期间，基于瑞士与坦桑尼亚就健康促进和系统强化项目开展的合作，该国制定并实施了一项改善坦桑尼亚联合共和国医疗器械管理系统的计划。通过开设新的培训课程和在政府中设立永久职位，涌现了越来越多的生物医学工程师。此外，还建造了更多的地区和区域维护和维修车间，并建立了一个国家校准和培训中心，以确保医疗器械的正常运行。电子医疗器械管理系统的引入为医疗机构和卫生部提供了与医疗器械运行状况以及维修和备件需求相关的数据。鼓励各级政府加大在医疗器械管理方面的人力和财力投入。经过长达十年的努力，正常运行设备的比例大幅增加，且设备成本也因进行维修而不是直接更换而有所降低。该项目还证明了将人员培养、维护和维修机构、文件编制和管理以及政府政策和预算情况纳入考虑范畴的综合系统强化方法的重要性。

## Резюме

Медицинские технологии играют центральную роль в повышении производительности и качества систем здравоохранения. Однако во многих странах Африки к югу от Сахары системы управления медицинскими изделиями слабы или вовсе отсутствуют. Цель данной статьи заключается в том, чтобы на конкретном примере показать, как политические реформы могут помочь обеспечить воплощение политики в области медицинских технологий в повседневную практику и как интегрированный системный подход может усовершенствовать процесс управления медицинскими изделиями. В период с 2011 по 2023 год в рамках швейцарско-танзанийского сотрудничества в ходе проекта по развитию здравоохранения и укреплению систем был разработан и реализован план по совершенствованию систем управления медицинским оборудованием в Объединенной Республике Танзания. Благодаря новым учебным курсам и созданию постоянных должностей в правительстве повысился уровень подготовки инженеров по медицинскому оборудованию. Кроме того, были построены дополнительные окружные и региональные мастерские по техническому обслуживанию и ремонту, а также создан Национальный центр калибровки и обучения для обеспечения правильного функционирования медицинских изделий. Внедрение электронной системы управления медицинскими изделиями позволило медицинским учреждениям и Министерству здравоохранения получать данные об эксплуатационном состоянии медицинских изделий и потребности в ремонте и запасных частях. Правительствам всех уровней было рекомендовано выделять больше человеческих и финансовых ресурсов на управление медицинскими изделиями. После проведения работ на протяжении десяти лет процент исправного оборудования значительно увеличился, а расходы сократились за счет ремонта, а не замены оборудования. В рамках проекта также была продемонстрирована ценность комплексного подхода к укреплению системы, который охватывал персонал, техническое обслуживание и ремонт, документацию и управление, а также государственную политику и бюджетное планирование.

## Introduction

Health-care technology includes medical devices, procedures and systems, as well as medicines and vaccines, and involves the application of organized knowledge and skills.^1^^–^^3^ Such technology plays a crucial role in addressing health needs and improving the quality of health services. However, in many countries in the African Region, the systematic management of medical devices faces particular challenges. First, there is a global shortage of biomedical engineers to repair malfunctioning devices.^4^^,^^5^ Second, as most health-care technology is imported, there is a mismatch between technology development in high-income countries and specific local needs,^6^ which affects the cost and utilization of the technology.^1^^,^^7^^–^^9^ Furthermore, in the absence of systematic planning and monitoring of medical equipment, countries may adopt multiple, similar solutions to dealing with technical problems, which can result in duplication and complicate the management of spare parts and repair services. According to reports, formal medical device management is either ineffective or lacking in Africa.^3^^,^^10^^–^^12^ For instance, in the United Republic of Tanzania (here we refer specifically to the mainland), there was virtually no system in place for medical device management before 2011.^13^ During the last decade, however, medical device management in the country has undergone changes thanks to a continuous Swiss–Tanzanian bilateral effort in cooperation with partners from the private sector and other development agencies, most notably the Korea International Cooperation Agency. Today, medical device management has been structurally integrated into the Tanzanian health ministry.

The purpose of this article is to illustrate, using a case study, how policy reforms can facilitate the transition from policy on health-care technology to everyday practice, and how an integrated systems approach can enhance operational effectiveness. Specifically, we describe the system changes developed cooperatively by the Tanzanian health ministry and the Health Promotion and System Strengthening Project (hereafter the project), which was funded by the Swiss government and implemented by the Swiss Tropical and Public Health Institute.^14^

## Policy environment

### Policy and practice in 2011

**Fig. 1:**
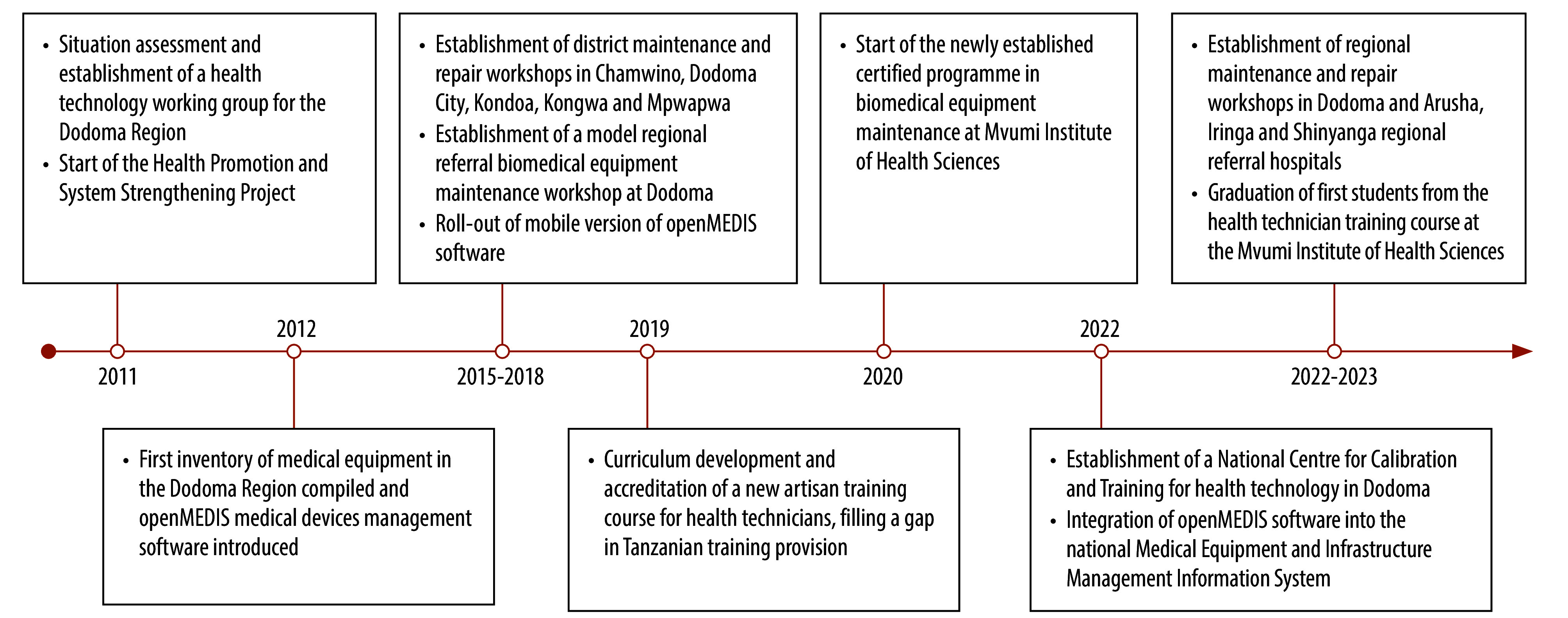
Milestones, medical device management reform, United Republic of Tanzania, 2011–2023

Note: Artisans are health-care staff who have undertaken an apprenticeship or received hands-on training in medical device maintenance.

In 2010, during the preparatory phase of the project, we (the health ministry and the project) conducted a situation assessment. The resulting report^13^ revealed that the 2007 National Health Care Technology Policy Guideline had still not been fully implemented (Fig. 1).^15^ Although the guideline outlined a referral system for planning and managing the maintenance of health-care technology, which included the establishment of zonal, regional and district workshops, implementation was poor and support from the health ministry was limited. Moreover, we found that there were few qualified technical staff for planning and maintaining medical devices, including: (i) biomedical engineers; (ii) technicians, who had undergone formal education and received a certificate from a technical school, community college or similar institution; and (iii) artisans, who had undertaken an apprenticeship or received more hands-on training. As there was no specific training in the country in 2011, engineers and technicians had to be trained abroad. Some technical personnel were available at the district level but were mostly lacking at the health facility level, and funding for maintenance was completely absent. In terms of infrastructure, most health-care facilities from primary to tertiary level lacked qualified staff to plan and execute maintenance activities. Although skilled artisans were available, maintenance needs were not adequately addressed by health-care facilities, district management or budgets. The lack of a maintenance culture was evident in the absence of a systematic approach, regular inspections and long-term maintenance planning. In addition, the supervision of outsourced services was often insufficient, which raised concerns about service quality.

The situation assessment was discussed at a planning workshop in 2010 that involved local government stakeholders, development partners and private institutions. As a result, a plan of action was devised and a health technology working group was established for the Dodoma Region to oversee the implementation of the plan of action and to maintain contact with national authorities and organizations (Fig. 1).^13^ The working group consisted of representatives from district and regional government health management teams, technical staff from districts and health facilities, and representatives of private and faith-based health facilities.

### Principles for a new policy

Based on our initial assessment, we took a holistic systems approach to reforming medical device management. We piloted the implementation of the medical device management system in the Dodoma Region, guided by four principles: (i) alignment with national technology management policy and regulations; (ii) utilization of all available maintenance resources, including those of local and central government, health facilities, the private sector and faith-based organizations; (iii) integration of the maintenance of both medical equipment and infrastructure into overall management of the health system and individual health-care facilities; and (iv) adaptability, scalability and sustainability of the suggested improvements.

We chose these four principles because, first, they reflect the project’s core values and its mandate to adopt a system-strengthening approach that supports the government health-care system and does not create an insular, fragmented and donor-dependent arrangement. Second, these principles were a pragmatic reaction to a situation in which human resources were scarce and where all available sources of knowledge had to be mobilized and integrated to construct a comprehensive medical device management system. Third, by integrating the maintenance of infrastructure with the maintenance of medical equipment into a single management system, it becomes possible to gain an overview of each health facility’s functioning and to ensure community-based organizations can manage and supervise local, small and labour-intensive maintenance activities.^16^

The development of the medical device management system followed established health-care technology management practices.^17^ In addition, tools were provided for documentation and reporting, such as: (i) standard operating procedures; (ii) data collection checklists for equipment inventories; (iii) planning and budgeting templates; and (iv) maintenance registers. These tools were digitized and integrated into the national Medical Equipment and Infrastructure Management Information System.

**Fig. 2:**
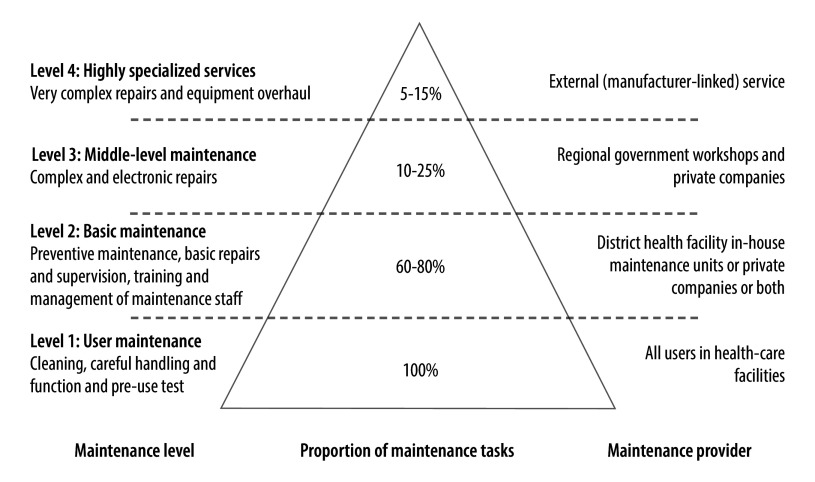
Maintenance pyramid for medical devices, United Republic of Tanzania

Adapted from Raab.^18^

The maintenance pyramid in Fig. 2 illustrates a sustainable maintenance system for medical devices. Our primary focus was on basic maintenance at district, municipal and health facility levels as well as on preventive maintenance by users, which correspond to levels 1 and 2 in the pyramid.

### Policy dialogue and guidelines

Within the framework of the project, the health ministry entered into a dialogue and collaboration on health-care technology policy with national authorities through its Diagnostic and Health Care Technical Services Section. All resulting actions were designed and discussed by the regional health technology working group established within the project and were endorsed for implementation by local government authorities. In addition, the working group explored the establishment of a reference and referral workshop at the regional level, and discussed strategies for strengthening personnel by situating biomedical engineers at regional and district levels. Systematic planning processes and budget allocations for health-care technology within local government were supported. Members of the working group also lobbied district administrations to raise awareness of medical device management and of the need for an earmarked maintenance budget. The establishment of a national inventory of medical equipment was facilitated by the project to guide the planning, budgeting, procurement and disposal of devices. In collaboration with the President's Office – Regional Administration and Local Government, we developed policy guidelines that provided a strategic direction for medical device management and that covered planning, acquisition, installation, inventory management, training, maintenance and decommissioning. The health ministry issued an operational manual to assist in the practical implementation of these guidelines.

### Policy development

The health ministry acknowledged the importance of medical device management in all its health sector strategic plans. Three plans illustrate how the importance and anchoring of strategic interventions grew: Health Sector Strategic Plan III (2009–2015) only touched briefly on medical device management,^19^ whereas Health Sector Strategic Plan IV (2015–2020) embedded medical device management within the infrastructure, transport and equipment strategy.^20^ Subsequently, Health Sector Strategic Plan V (2021–2026) outlined specific strategic outcomes for health infrastructure and committed to improved preventive maintenance.^21^ At the time of writing, the government intended to create a new long-term investment plan for health facilities that aimed: (i) to strengthen planned preventive maintenance of buildings and equipment; and (ii) to develop guidelines for the staffing of technicians in health facilities, which will ensure sufficient technical personnel are available for repair and maintenance services. The government’s current scheme of services aims for the following technical staffing levels: (i) one technician and one artisan in each health centre; (ii) one technician and four artisans in each district hospital; and (iii) one biomedical engineer, four technicians and 10 artisans in each regional hospital.

### Institutional changes

Due to economic constraints, dependence on donors and inadequate implementation of the National Health Policy, the Tanzanian government has faced problems with simultaneously maintaining various pieces of technology with different brands. In the 1970s, the government attempted to rectify this situation by introducing and establishing equipment maintenance systems. The health ministry established hospital maintenance workshops at referral hospitals in the cities of Dar es Salaam, Mbeya, Moshi and Mwanza. Further, maintenance workshops were established to serve voluntary agency hospitals under the Christian Social Services Commission. In addition, personnel from workshops belonging to the Ministry of Works, Transport and Communications visited hospitals when requested. However, these personnel did not have the expertise in medical device technology required. More specialized services, such as the repair and maintenance of X-ray equipment, were contracted to private companies.

In 2011, the Director of Curative Services of the health ministry oversaw the medical device management through the Diagnostic and Health Care Technical Services Section. In 2022, the health ministry underwent reforms, which led to the establishment of a new Directorate of Diagnostic and Health Care Technical Services,^22^ comprised of three main components: (i) radiology and imaging services; (ii) health laboratory services; and (iii) health-care technical services. This transition from a section to a directorate markedly enhanced the importance of medical device management within the ministry and was accompanied by a corresponding additional allocation of personnel and funding.

## The systems approach

The reforms initiated by the health ministry within the cooperation framework of the project led to the adoption of a systems approach to addressing problems with medical device management. Box 1 describes the components of this approach.

Box 1 **Approaches to addressing problems with medical device management, United Republic of Tanzania**

1. Building human resource capacity through on-the-job training and the establishment of a new diploma course in medical engineering, thus filling gaps in existing training programmes;
2. Building repair and maintenance capabilities at district and regional workshops;
3. Providing management instruments, such as a medical equipment and infrastructure management information system; and
4. Providing support for medical device management through integration into the responsibilities of council health management teams at district and municipal levels and through policy and budget adjustments.

### Human resources

We reformed the human resource sector in accordance with the maintenance pyramid (Fig. 2).^18^ In doing so, we involved users, health facilities and district and regional government, and considered policy changes. Initially, managerial capacity was enhanced through a series of short training courses for existing and new biomedical technicians on management skills, planning, budgeting and medical device process management.^23^ In addition, the health ministry established strategic technical development plans at district and health facility levels to improve the procurement, maintenance, repair and extended use of medical devices as well as the renovation of buildings, while ensuring efficient use of budgets. We further bolstered technician capacity by defining the profile of a district technician; this enabled systematic skills development, peer-to-peer training, and the development of a curriculum for a competency-based certificate programme in biomedical equipment maintenance at the Mvumi Institute of Health Sciences in 2020 (Fig. 1).^24^ Higher-level personnel were also recruited, including biomedical engineers and technicians at district and municipal levels and maintenance coordinators at the regional level. User capabilities were increased through the provision of community-based repair and maintenance mechanisms, standard operating procedures and on-site training for health-care workers. Biomedical engineers and technicians were integrated into council health management teams at district and municipal levels and into regional health management teams, thereby ensuring that medical device management was considered during health policy discussions.

**Fig. 3:**
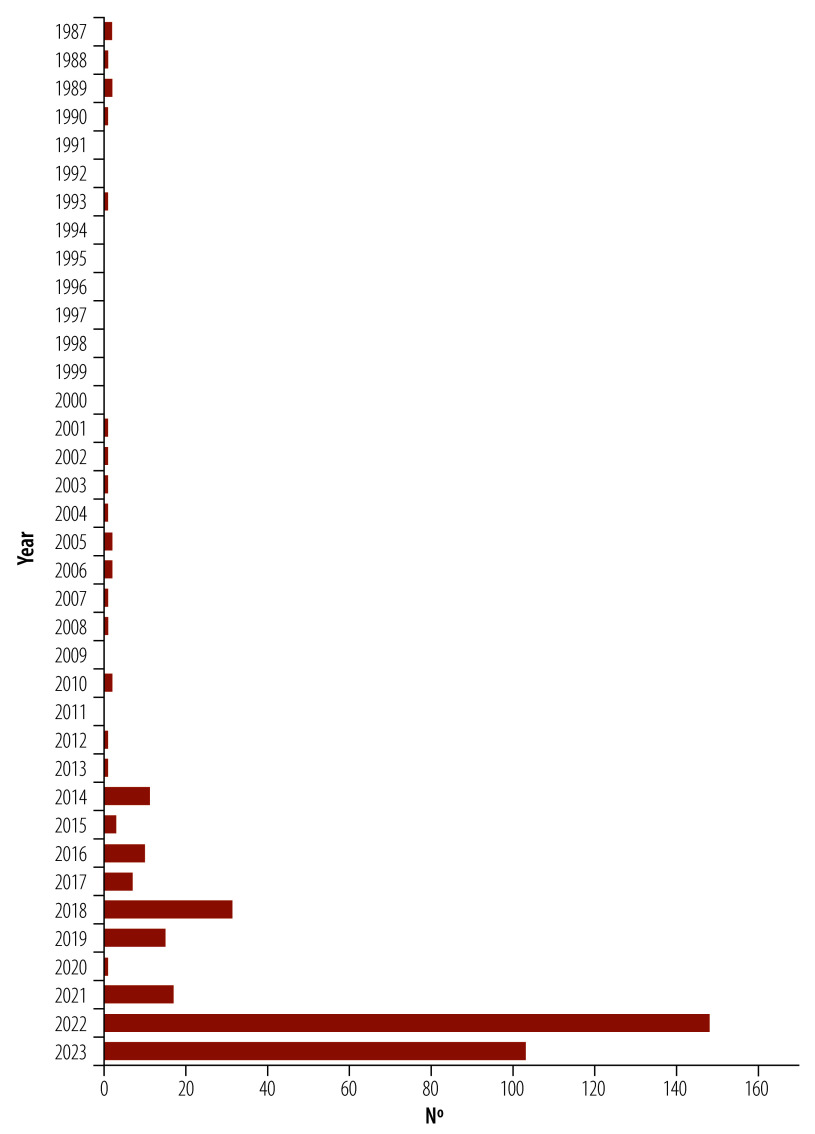
Additional biomedical equipment technicians permanently employed each year, United Republic of Tanzania, 1987–2023

These reforms complemented technician training initiated by the government in 2011 at the Dar es Salaam Institute of Technology and the Arusha Technical College and subsequently expanded to other institutions. These institutions offered courses at levels ranging from competency-based certificates to academic degrees, as defined by the United Republic of Tanzania’s qualification framework.^25^ Notably, the government substantially increased the number of biomedical engineering professionals between financial year 2014 to 2015, when the first technicians graduated, and financial year 2022 to 2023, such that the total number employed rose to 367 permanent positions and 39 contract staff. This increase is illustrated by the annual rise in new permanent employment positions for biomedical engineering professionals shown in Fig. 3. The high number in 2022 reflects the health ministry’s endeavours to deal with a shortage of personnel. Subsequently, the number of new positions fell slightly in 2023.

### Repair and maintenance

**Fig. 4:**
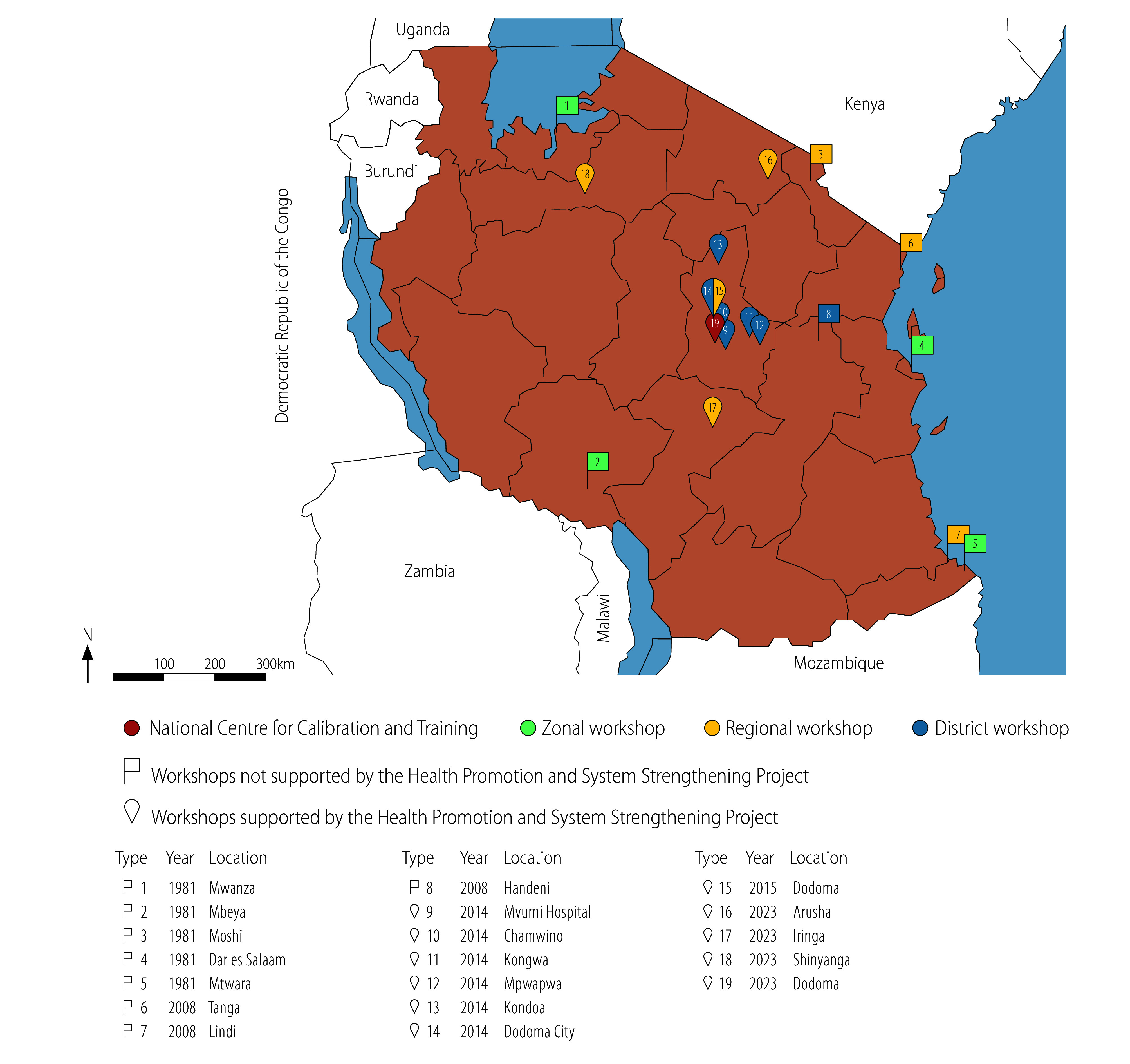
Medical device maintenance workshops operated by the health ministry, United Republic of Tanzania, 2023

Notes: Only workshops on mainland United Republic of Tanzania are shown. For effective implementation, the government divided the country into seven zones: Central, Coastal, Lake, Northern, Southern, Southern Highlands and West Lake. The health ministry established maintenance and repair workshops in some of these zones, each of which serviced several administrative regions. In Dodoma, the Health Promotion and System Strengthening Project supported two workshops: a district-level maintenance and repair workshop operated by Dodoma City Council (2014) and a model regional referral biomedical equipment maintenance workshop at the regional referral hospital (2015). In addition, the National Centre for Calibration and Training was established at the Mirembe Institute of Mental Health at Dodoma in 2022.

In 2011, there were limited repair and maintenance operations for medical devices at four zonal workshops in Dar es Salaam, Mbeya, Mtwara and Mwanza. Regional workshops existed in Lindi, Moshi and Tanga, and there were workshops at district hospitals in Handeni and Mvumi. The workshop at the faith-based Mvumi Hospital was established in 1993 and refurbished with the support of the project in 2014, when it was integrated into the health ministry’s system. Within the framework of the project, we also established maintenance and repair workshops at district hospitals in Chamwino, Dodoma City, Kondoa, Kongwa and Mpwapwa, and equipped them with high-quality maintenance and repair tools (Fig. 4). Between 2015 and 2023, workshops were completed in five districts and four regions. Notably, a model regional referral biomedical equipment maintenance workshop was constructed in Dodoma in 2015, which demonstrated best maintenance practices at the regional level. This construction was followed by workshops in regional referral hospitals in Arusha, Iringa and Shinyanga (Fig. 4). In addition, administrative tools were introduced to support repair and maintenance, including: (i) forms for reporting technical problems; (ii) job cards for repairs; (iii) equipment history cards for tracking performance; (iv) forms for the return of repaired equipment that included user instructions; and (v) openMEDIS open-source software (Swiss Tropical and Public Health Institute, Basel, Switzerland) for inventory data entry and recording.

### Information technology 

We incorporated openMEDIS software into the government’s Medical Equipment and Infrastructure Management Information System (MEIMIS) in 2022 (Fig. 1). This management information system was made available nationwide by the health ministry with the support of the project. Currently, the system facilitates inventory management for all types of health-care facility, including hospitals, health centres and dispensaries. The system also aids equipment management and supports informed decision-making on investment, technology deployment, maintenance and decommissioning. Having an online register of the operational status of all equipment greatly aids the government in managing the medical devices nationwide.

### National support structure

In December 2022, the health ministry established the National Centre for Calibration and Training at the Mirembe Institute of Mental Health at Dodoma to meet the increasing demand for calibration services.^26^ This is the only facility in the country for calibrating medical equipment and for providing in-service training to biomedical engineers and technicians (Fig. 1). The centre ensures repaired equipment meets safety standards, performs reliably and accurately, and complies with the manufacturers’ specifications. During preparatory work for the centre in 2019, the project helped the health ministry identify the tools and equipment needed, develop product specifications and carry out procurement. The procurement of some highly specialized equipment was not feasible within the project's timeframe and further funding was subsequently obtained by the health ministry from, for example, the Kingdom of the Netherlands, the Republic of Korea and Unitaid.

### Cost of maintenance systems

In 2018, we assessed the financial cost of establishing maintenance systems during an evaluation of our medical device management interventions.^23^ We found that the average annual cost of operating a fully functioning medical device management system for a district, including a workshop with technicians serving all district health facilities, was approximately 20 000 United States dollars (US$). However, if national policy allows for some costs to be omitted due to scaling (e.g. the availability of adequate, central, training programmes that render short-term training schemes redundant) and routine procedures (e.g. the availability of centralized guidelines that render some decision-making processes redundant), the average annual budget could be reduced to US$ 10 000 to US$ 15 000.

### Future developments

An evaluation of our medical device management interventions demonstrated that substantial progress has been made. In surveys, the proportion of buildings and infrastructure inspected by technicians each year rose from 34% (81/241) in 2011 to 69% (79/114) in 2018, and the proportion of medical equipment in health facilities inspected increased from 35% (85/241) to 83% (94/114) over the same period.^23^ Moreover, 73% (83/114) of health facilities surveyed in 2018 had budgets for maintenance and repair allocated by their respective districts compared with 54% (122/228) in 2011.^23^ Despite this progress, challenges persist. For example, maintenance capacity is limited and there are problems with coordination. Nevertheless, a maintenance culture has been internalized within the health system, and health-care facilities now have annual plans and budgets for preventive maintenance.

In 2023, the health ministry, supported by the project, completed the construction and equipping of three additional regional referral workshops in Arusha, Iringa and Shinyanga, based on the model regional referral workshop established in Dodoma in 2015. Earlier across the country in 2020, regional governments demonstrated their strong commitment to medical device management by setting up maintenance workshops or rooms and by implementing inventory systems, all of which garnered considerable political support. The locations of all medical device maintenance workshops operated by the health ministry in 2023 are shown in Fig. 4. Nevertheless, there are still difficulties with coordination. Although coordination through the ministry improved following organizational restructuring and the creation of the new Directorate of Diagnostic and Health Care Technical Services, additional human resources and an increased budget allocation are needed from President's Office – Regional Administration and Local Government and the health ministry. In fact, government investment in human resources for maintenance services has increased markedly: between 2021 and 2022, over 150 additional biomedical engineers and technicians were employed nationally, (Fig. 3) and the national capacity to produce biomedical engineering specialists improved with the establishment of the Mvumi Institute of Health Sciences course, which can train 100 graduates annually. The first students graduated in 2023 and the course is being upgraded to a Diploma in Biomedical Engineering from the 2024 academic year.

Considerable challenges persist, for example, data entry into the management information system is often incomplete. In particular, gaps in repair data can hinder proper analysis and decision-making. Convincing district governments to allocate resources for medical device management and spare part procurement remains difficult. Moreover, the limited capacity of council health management teams and a lack of guidance from these teams present obstacles to implementing plans of action and to raising budgets for repair and maintenance. Constraints on recruitment also impede performance because the abilities of existing district technicians to learn and adopt new skills and expertise vary. Additionally, policy guidelines on medical device management still await final approval from the health ministry, and initial progress in providing and scaling-up budgets has been slow.

## Conclusions

The decade-long effort to strengthen and support the regional and national infrastructure for medical device management and operations in the United Republic of Tanzania we describe illustrates the importance and value of adopting an integrated systems approach. By recognizing the significance of maintenance and repair services for the overall functioning of the health sector, the country’s government succeeded in establishing a maintenance pyramid within the health system. This approach involved building technical capacity at all levels, from the health-care personnel operating equipment to biomedical technicians and engineers at repair workshops. The interventions introduced for infrastructure, training and policy laid the foundations for this achievement.

The system-strengthening approach we adopted was also important for simultaneously addressing challenges at interlinked levels: (i) the trained technicians and biomedical engineers essential for planning and maintenance; (ii) the district and regional workshops required for repair and maintenance; (iii) the information technology management tools vital for information and task coordination; and (iv) the anchoring of key operational strategies within government policies and budgets crucial for sustainable medical device management.

Our systems approach required innovations to progress through three stages to ensure sustainability, including developing interventions, optimizing service delivery systems and transforming institutional policies. Innovations, such as a new information technology equipment management system, were developed within the project’s framework on the basis of local assessments and international best practice. During the second stage, these innovations had to be integrated into broader service delivery systems, such as the national policy framework for information technology and health facilities’ information technology systems. Finally, after testing, adjustment and approval, innovations had to be integrated into government policy and individual institutions in the form of guidelines, policy documents and budget allocations. Each stage from innovation to integration to policy adaptation required cooperation at multiple levels and the commitment of human and financial resources.

Looking ahead, national rollout of the innovations we developed promises to strengthen medical equipment management in the country. Key milestones were establishing a National Centre for Calibration and Training and building the model regional referral biomedical equipment maintenance workshop in Dodoma. In developing an infrastructure for medical device management, addressing technical and political challenges and enhancing coordination throughout the country is crucial. Establishing a national policy and securing funding commitments are decisive factors.

## References

[1] Houngbo PT, Zweekhorst M, Bunders J, Coleman HLS, Medenou D, Dakpanon L, et al. The root causes of ineffective and inefficient healthcare technology management in Benin public health sector. Health Policy Technol. 2017;6(4):446–56. 10.1016/j.hlpt.2017.06.004 PMC5627786 28949474

[2] Houngbo PT, Coleman HLS, Zweekhorst M, De Cock Buning T, Medenou D, Bunders JFG. A model for good governance of healthcare technology management in the public sector: learning from evidence-informed policy development and implementation in Benin. PLoS One. 2017 Jan 5;12(1):e0168842. 10.1371/journal.pone.0168842 28056098 PMC5215885

[3] Houngbo PT, De Cock Buning T, Bunders J, Coleman HLS, Medenou D, Dakpanon L, et al. Ineffective healthcare technology management in Benin’s public health sector: the perceptions of key actors and their ability to address the main problems. Int J Health Policy Manag. 2017 Oct 1;6(10):587–600. 10.15171/ijhpm.2017.17 28949474 PMC5627786

[4] Inagaki D, Nakahara S, Chung UI, Shimaoka M, Shoji K. Need for improvements in medical device management in low- and middle-income countries: applying learnings from Japan’s experience. JMA J. 2023 Apr 14;6(2):188–91. 10.31662/jmaj.2022-0089 37179730 PMC10169262

[5] Global strategy on human resources for health: Workforce 2030. Geneva: World Health Organization; 2016. Available from: https://www.who.int/publications/i/item/9789241511131 [cited 2024 May 22].

[6] Elsenhans H. Der Mythos der Kapitalintensität und die notwendige falsche Technologiewahl der Entwicklungsländer. In: Kohler-Koch B, editor. Technik und internationale Entwicklung. Baden-Baden: Nomos; 1986. pp. 267–90. German.

[7] Aqil A, Lippeveld T, Hozumi D. PRISM framework: a paradigm shift for designing, strengthening and evaluating routine health information systems. Health Policy Plan. 2009 May;24(3):217–28. 10.1093/heapol/czp010 19304786 PMC2670976

[8] Heeks R. Information systems and developing countries: failure, success, and local improvisations. Inf Soc. 2002;18(2):101–12. 10.1080/01972240290075039

[9] Lustick DR, Zaman MH. Biomedical engineering education and practice challenges and opportunities in improving health in developing countries. In: Proceedings, 2011 Atlanta Conference on Science and Innovation Policy; 2011 Sep 15–17; Atlanta, United States of America. New York: IEEE; 2011. 10.1109/ACSIP.2011.6064477

[10] Temple-Bird C. Managing the import and use of healthcare technology in sub-Saharan Africa [PhD thesis]. Milton Keynes: The Open University; 2005.

[11] Kachieng’a MO. Health technology assessment in sub-Saharan Africa: a cross-national study of Kenya and South Africa [PhD thesis]. Capetown: University of Capetown; 1999.

[12] Baseline country survey on medical devices 2010. Geneva: World Health Organization; 2011. Available from: https://www.who.int/publications/i/item/WHO-HSS-EHT-DIM-11.01 [cited 2024 Apr 17].

[13] Werlein R, Haule S. Report situation analysis HPSS Dodoma Tanzania. Health technology management. Basel: Swiss Tropical and Public Health Insitute; 2011. Available from: https://www.hpss.or.tz/_files/ugd/c2281e_1e554873109f44d2839a89c6ed79f60c.pdf [cited 2024 Aug 12].

[14] Health Promotion and System Strengthening (HPSS) Project. Strengthening the health system in Tanzania. HPSS-Tuimarishe Afya Project [internet]. Dodoma: Health Promotion & System Strengthening (HPSS) Project; 2022. Available from: https://www.hpss.or.tz/ [cited 2024 May 22].

[15] Sera ya afya [National health policy]. Dodoma: United Republic of Tanzania Ministry of Health and Social Welfare; 2007. Swahili. Available from: https://hssrc.tamisemi.go.tz/storage/app/uploads/public/5ac/f1c/e8a/5acf1ce8a80ef622965601.pdf [cited 2023 May 22].

[16] Meshack M. Potential and limitations of stakeholders’ participation in community-based projects: the case of Hanna Nassif roads and drains construction and maintenance in Dar es Salaam, Tanzania. Int Dev Plan Rev. 2004;26(1):61–82. 10.3828/idpr.26.1.4

[17] Lenel A, Temple-Bird C, Kawohl W, Kaur M. Guide 1. How to organize a system of healthcare technology management. Lewes: Ziken International (Consultants) Ltd; 2005. Available from: https://f.hubspotusercontent30.net/hubfs/8702981/HCT%20Guide%201%20-%20How%20to%20Organize%20a%20System%20of%20Healthcare%20Technology%20Management.pdf [cited 2024 Feb 29].

[18] Raab M. Maintenance strategies. Basel: Swiss Tropical and Public Health Institute; 1999.

[19] Health Sector Strategic Plan III July 2009 – June 2015: partnership for delivering the MDGs. Dodoma: United Republic of Tanzania Ministry of Health and Social Welfare; 2009. Available from: https://extranet.who.int/countryplanningcycles/sites/default/files/country_docs/Tanzania/ndp_tanzania.pdf [cited 2023 May 22].

[20] Health Sector Strategic Plan July 2015 – June 2020 (HSSP IV): reaching all households with quality health care. Dodoma: United Republic of Tanzania Ministry of Health and Social Welfare; 2015. Available from: https://www.prb.org/wp-content/uploads/2020/06/Tanzania-Health-Sector-Strategic-Plan-IV-2015-2020-1-4.pdf [cited 2023 May 22].

[21] Health Sector Strategic Plan July 2021 – June 2026 (HSSP V): leaving no-one behind. Dodoma: United Republic of Tanzania Ministry of Health, Community Development, Gender, Elderly and Children; 2021. Available from: https://extranet.who.int/countryplanningcycles/sites/default/files/public_file_rep/TZA_Tanzania_Health-Sector-Strategic-Plan-V_2021-2026.pdf [cited 2023 May 22].

[22] The approved functions and organisations of the Ministry of Health. Approved by the President on 4th July, 2022. Dodoma: President’s Office of Public Service Management and Good Governance, United Republic of Tanzania: 2022.

[23] Abdallah AK, Haule S, Ruhago G, Werlein R. Progress and impact evaluation of the health technology management interventions of the HPSS Project. November 2018 – April 2019. Basel: Swiss Tropical and Public Health Institute; 2019. Available from: https://www.hpss.or.tz/_files/ugd/c2281e_fc13baf2a8094c0d816dcbe6c9c430a9.pdf [cited 2024 May 21].

[24] Biomedical engineering [internet]. Mvumi Mission: Mvumi Institute of Health Sciences; 2024. Available from: https://mihs.ac.tz/program-post/faculty-of-engineering/ [cited 2023 May 21].

[25] The National Council for Technical and Vocational Education and Training (NACTVET) [website]. Dodoma: The National Council for Technical and Vocational Education and Training, United Republic of Tanzania; 2024. Available from: https://nactvet.go.tz [cited 2023 May 21]

[26] Concept note for the establishment of the National Medical Equipment Calibration and Training Center. Dodoma: Ministry of Health, Community Development, Gender, Elderly and Children, United Republic of Tanzania; 2021. Available from: https://51f4f53b-871b-437c-9daf-803b92ec96de.filesusr.com/ugd/c2281e_7182f371b3404b8cab2784f7d9e9a1cb.pdf [cited 2023 May 22].

